# Direct observation of DNA alterations induced by a DNA disruptor

**DOI:** 10.1038/s41598-022-10725-8

**Published:** 2022-04-28

**Authors:** Takahito Ohshiro, Ayumu Asai, Masamitsu Konno, Mayuka Ohkawa, Yuki Komoto, Ken Ofusa, Hideshi Ishii, Masateru Taniguchi

**Affiliations:** 1 SANKEN (The Institute of Scientific and Industrial Research), Osaka University, 8-1 Mihogaoka, Ibaraki, Osaka 567-0047 Japan; 2 Artificial Intelligence Research Center, SANKEN (The Institute of Scientific and Industrial Research) Osaka University, 8-1 Mihogaoka, Ibaraki, Osaka 567-0047 Japan; 3 Center of Medical Innovation and Translation Research, Graduate School of Medicine, Osaka University, 2-2 Yamadaoka, Suita, Osaka 560-0085 Japan; 4 Division of Tumor Biology, Research Institute for Biomedical Sciences (RIBS), Tokyo University of Science, 2641 Yamazaki, Noda, Chiba 278-8510 Japan; 5 Prophoenix Division, Food and Life-Science Laboratory, Idea Consultants, Inc., 1-24-22 Nanko-kita, Suminoe-ku, Osaka-City, Osaka 559-8519 Japan; 6 Present Address: SANKEN (The Institute of Scientific and Industrial Research), Osaka University, 8-1 Mihogaoka, Ibaraki, Osaka 567-0047 Japan

**Keywords:** Analytical biochemistry, Genomic analysis, Nanoscience and technology

## Abstract

DNA alterations, such as base modifications and mutations, are closely related to the activity of transcription factors and the corresponding cell functions; therefore, detection of DNA alterations is important for understanding their relationships. Particularly, DNA alterations caused by exposure to exogenous molecules, such as nucleic acid analogues for cancer therapy and the corresponding changes in cell functions, are of interest in medicine for drug development and diagnosis purposes. However, detection of comprehensive direct evidence for the relationship of DNA modifications/mutations in genes, their effect on transcription factors, and the corresponding cell functions have been limited. In this study, we utilized a single-molecule electrical detection method for the direct observation of DNA alterations on transcription factor binding motifs upon exposure to a nucleic acid analogue, trifluridine (FTD), and evaluated the effects of the DNA alteration on transcriptional activity in cancer cell line cells. We found ~ 10% FTD incorporation at the transcription factor p53 binding regions in cancer cells exposed to FTD for 5 months. Additionally, through single-molecule analysis of p53-enriched DNA, we found that the FTD incorporation at the p53 DNA binding regions led to less binding, likely due to weaken the binding of p53. This work suggests that single-molecule detection of DNA sequence alterations is a useful methodology for understanding DNA sequence alterations.

## Introduction

DNA alterations, such as base modifications or substitution (mutations), are of interest because they are closely related to various biological phenomena, such as cell differentiation and diseases. Since these phenomena are mediated by transcription factors^1,2^, it is considered that DNA alterations affect the activity of transcription factors and corresponding cell functions^3–5^. Particularly, DNA alterations by exogenous DNA disruptors, such as nucleic acid analogs for cancer therapy and environmental pollutants, get attention because they can induce nucleotide sequence alterations and corresponding malfunction of transcription factors due to altered transcription factor binding sites^6^. To understand the relationship between DNA modification/mutation, transcription factors, and cell function, it is important to comprehensively detect DNA alterations, including the identities of the base species altered and their positions in the DNA sequence. In this vein, high performance liquid chromatography (HPLC) and radioactive labeling have previously observed the incorporation of a nucleic-acid analogue into DNA^7^.

Single-molecule quantum measurement method has been a promising method for the simultaneous identification of the types of DNA modifications and/or mutations and their position in the DNA sequence. Since single-molecule quantum measurements can detect the physical properties of a sample nucleotide in the sample sequence, it can detect the location of any base alteration without polymerase chain reaction amplification^8–11^. When individual nucleotides of the sample DNA chain pass between the nanogap sensor electrodes, a tunnel phenomenon induces electron transfer through each nucleotide in sequence, resulting in the detection of electrical conductivity of individual nucleotides due to the unique electronic state of each nucleotide^12–19^. Therefore, the obtained signals represent characteristic conductance of small biomolecules, such as nucleotides and oligonucleotides^15,16^, artificial nucleotides^17^, amino acids^18^, and neurotransmitters^19^. Consequently, when a modification or mutation occurs in a DNA sequence, this method can detect and identify altered base species and their positions and count them at the single-molecule level.

**Figure 1:**
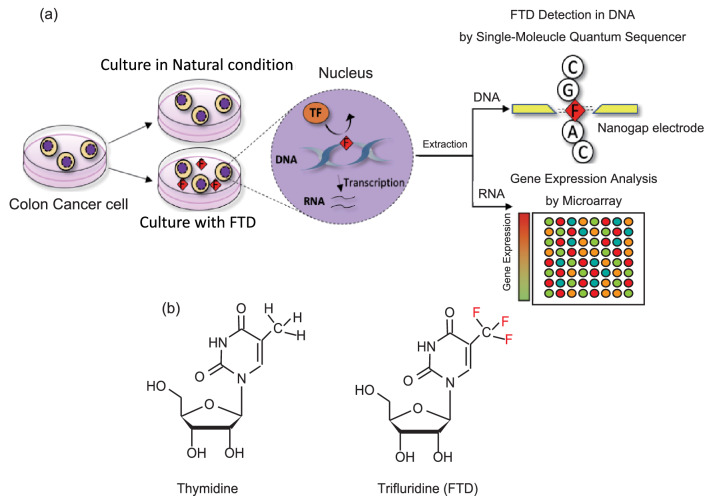
Flowchart of sequence-based analysis with exposing a DNA disruptor. (**a**) To evaluate the relationship between the conversion of DNA disruptors in DNA and cellular function, colon cancer cells were incubated with FTD, followed by DNA and RNA extraction. The DNA was sequenced by single-molecule sequencing to identify FTD conversion sites in DNA, and the RNA was analyzed using microarray to evaluate effects on transcription factor activity and cellular function. F and FTD; trifluridine, TF; transcription factor. (**b**) Chemical structure of thymidine (left) and FTD (right).

In this study, we investigated by single-molecule quantum detection the DNA alterations caused by an exogenous DNA disruptor and evaluated the effect on the activity of transcription and related cell function (Fig. 1a). As the DNA disruptor, we utilized a trifluridine (FTD) (Fig. 1b) on colon cancer cells. Since FTD is a nucleic acid analogue used as a cancer drug for colorectal cancer, it is expected that FTD can affect the transcription of cancer-related genes, such as apoptosis-related genes, through the alteration of *cis*-regulatory elements controlling such genes. The gene for *TP53* (encoding p53), a tumor suppressor gene and a regulator of cell death and apoptosis, suffers somatic mutations during carcinogenesis, many of which are loss-of-function mutations resulting in cell-death and apoptosis-related malfunction. However, not all cancers house mutations in the *TP53* gene, raising the possibility that wild-type *TP53* cancers acquire p53 dysfunction through the course of disease or therapy by alterative means, through mutations in the p53 binding sites of target genes. Conversely, it has not been known that FTD is incorporated into DNA and what effect it has on transcription factors and cell functions. In this study, we found around 10% of thymidine were substituted by FTD incorporation at the p53-binding regions upon FTD exposure in vitro. Additionally, by signal analysis of the immunoprecipitated DNA using anti-p53 antibody followed by single-molecule detection, we found that FTD incorporation at the DNA-binding regions weakened likely p53 binding. This technique allows for the first time the direct detection of DNA disruptors in DNA and allows for greater understanding of functions of DNA disruptors by linking molecular-level measurements to cognate biological effects.

## Results and discussion

### Detection of FTD incorporation instead of thymidine (dT) in p53-binding motifs in colorectal cancer cells

We performed single-molecule measurements for target DNA (Table 1) that are DNA binding regions of the p53 transcription factors extracted from cancer cell lines. The *TP53* gene is the most frequently mutated gene in human cancers with > 50% prevalence and contributes to preventing cancer formation as a tumor suppressor^20^; therefore, it is expected that, in the target DNA, DNA alterations have occurred under exposure to the nucleic acid analogue trifluridine (FTD). It has been reported that FTD exposure activates the p53 pathway and induces apoptosis^21,22^. We used two commonly used colon cancer cell lines, RKO and HCT-116, under FTD exposure or control conditions. To evaluate the effect of FTD exposure on the cancer cells, we prepared three experimental groups as following: cells without FTD exposure (P), cells with 1 month FTD exposure (F1), and cells with 5 months FTD exposure (F5).

**Table 1:** DNA sequence for target DNA and probe DNA.

DNA name	DNA sequence
p53 consensus sequence	5′-AXX CAT GCC CAX XCA TGC CC-3′
Target sequence	5′-AGA CAT GCC CAG ACA TGC CC-3′
Probe sequence (complementary target sequence)	5′GG GCs TGY YTG GGC sTG YYT-3′

*S* Abasic site, *X* A or G, *Y* C or T.

To capture the p53-binding motif, we utilized the binding consensus sequence of TP53 (upper row), which was previously reported^21^. The target sequence is set to be 5′-AGA CAT GCC CAG ACA TGC CC-3′ (middle row) in this study. Actually, the p53 recognition sites consist of two half-sites, which are separated by various length of sequences from 0 to 13 nucleotides. Since the spacer sequences vary; of 288 sequence samples, 236 (82%), 27 (9%) and 25 (9%) have 0, 1, and more spacers^23^, we utilized capture oligos with abasic-site spacers because they could cover significantly in total of p53 recognition sites based on the pervious study^23^. Therefore, to capture the target DNA, we designed the sequence (5′-GG GCs TGY YTG GGC sTG YYT-3′) as this probe DNA, where s (abasic site), X (A or G), and Y (C or T) in the probe sequence are used to provide redundancy in probe selectivity. Importantly, the abasic site is potential target of thymine (T) and its fluorinated thymine (FTD: trifluridine) in order to avoid forming any hydrogen bonds with T or FTD. As the abasic site, we utilized tetrahydrofuran-type abasic sites (1′,2′-dideoxyribose), which is called as “dSpacer”, for the spacer of capture sequence.

The experimental setup (Fig. 1a) involved lysing and extracting DNA in the first step. In the second step, the binding regions of p53 were captured and purified by the probe DNA complementary to the consensus p53-binding sequence (Table 1). From previous reports^23^, the consensus binding region of p53 is known to be 5′-AXX CAT GCC CAX XCA TGC CC-3′ (X = A or G). Based on the consensus sequence of p53, the present target sequence is set to be 5′-AGA CAT GCC CAG ACA TGC CC-3′. Since the chemical structure of FTD resembles thymidine (dT) (Fig. 1b), it is expected that FTD substitutions potentially occur at the number sixth position T (#6:T) and sixteenth position T (#16:T) within the consensus-binding region. In the third step, the conductivity of each captured DNA is measured by sequentially reading across individual single-nucleotides with nanofluid integrated nanogap devices (Fig. 1), in which the nanofluid can strongly confine nucleotide translocation, resulting in the straight-guiding of the nucleic acid molecules into the fluid region under DC voltage across the gap-electrode. The resulting conductance–time profiles represent the conductance sequence of each nucleotide in synthesized oligonucleotides translocating through the gap-electrodes (SI: S1 and Fig. S1). In the fourth step, by using a Phred base-calling method (SI: S2 and S3), we performed signal analysis, including picking-up and base-calling for each conductance–time profile based on the conductivity of all kinds of potential nucleotides including FTD mono-nucleotide and determined the sequences of the oligonucleotides translocating through the gap-electrodes. The conductance values order was as follows: dG (87 pS) > dA (67 pS) > dC (60 pS) > dT (36pS) > FTD (18 pS) (Table 2)^15,17^. In the final step, these determined sequences were mapped by assembling against an original sample sequence (SI: S3). Based on this mapped sequence, the conductance profiles were obtained and the FTD conversion rate in the sample nucleotide is evaluated, especially for each thymine site in the sample nucleotides (SI: S7).

**Table 2:** Single-molecule conductance and relative single-molecule conductance of deoxyribonucleosides and trifluridine (FTD).

DNA	Deoxyribose nucleoside name	Conductance (pS)	Relative *G*
G	Guanosine	86.7	1
A	Adenosine	66.8	0.77
C	Cytidine	59.5	0.69
T	Thymidine	39.1	0.45
FTD	FTD monophosphate	17.9	0.21

Single-molecule conductance was obtained from the peak of conductance histograms as previously described^15,17^.

**Figure 2:**
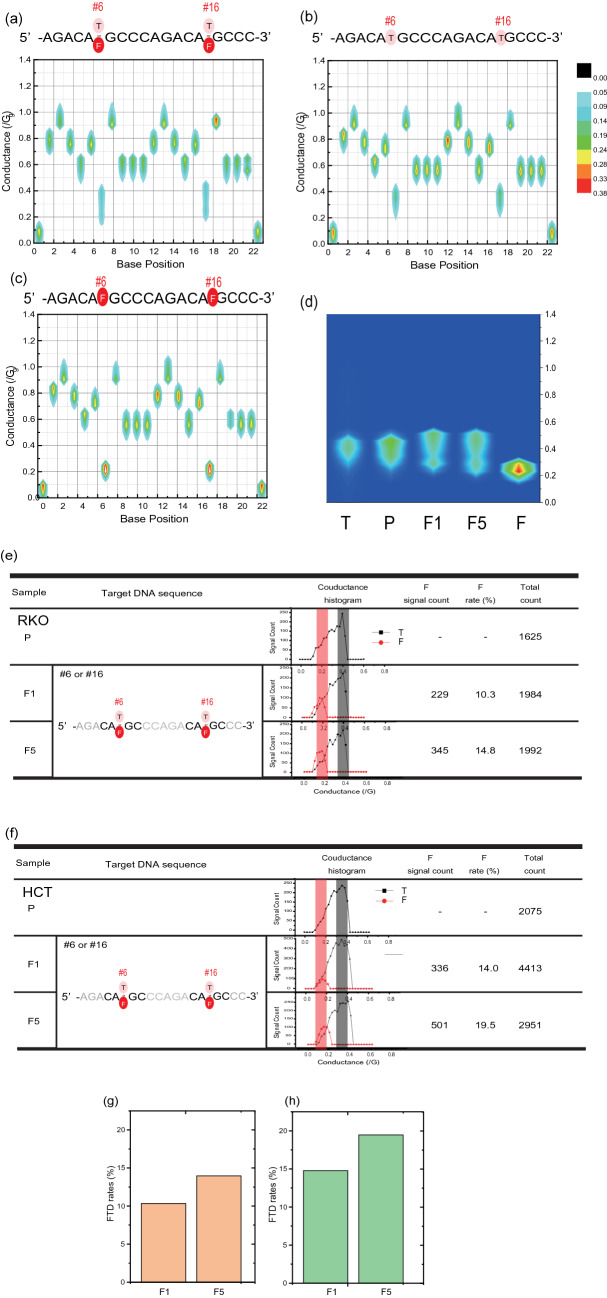
Conductance plots for FTD-incorporated DNA of transcription factor, p53, binding regions. (**a**) Heat maps of DNA conductance plots of DNA motif for p53 binding extracted from colorectal cancer line RKO (P). (**b**) Heat maps of synthesized DNA motif for p53 binding, wherein #6 and #16 thymines are non-fluorinated. (**c**) Heat maps of DNA conductance plots of synthesized DNA motif for p53 binding wherein #6 and #16 thymines are fluorinated. The x and y axes are the base position and conductance normalized to the conductance of guanine, respectively. (**d**) Enlarged conductance plots of the #16 position thymine for non-fluorinated (left), captured DNA motif for p53 binding for P (second left), captured DNA motif for p53 binding for F1 (middle), captured DNA motif for p53 binding for F5 (second right), and fluorinated DNA motif for p53 binding (right). (**e**, **f**) show each FTD incorporation rate in p53-binding motif DNA for RKO and HCT-116 cell lines, respectively. In the second column, the sequences neighboring of the FTD-incorporated position (#6, #16) are shown. In the third column, the conductance histograms relative to those of guanine are shown. The black and red lines represent the typical relative conductance values for T and FTD, respectively (Table 1). (**g**) FTD-exposure time dependency on FTD incorporation rates for RKO. (**h**) FTD-exposure time dependency on FTD conversion rates for HCT-116. FTD, trifluridine.

First, we obtained conductance signals from the RKO cell line cells for each experimental group. Each signal was reassembled based on the p53-binding motif sequence (5′-AGA CAT (or F) GCC CAG ACA T (or F) GC CC-3; F = FTD) to obtain a conductance plot. Figure 2a shows conductance plots for DNA extracted from RKO cancer cells (F5 sample), while conductance plots are shown for synthesized target non-FTD oligonucleotide (5′-AGA CAT GCC CAG ACA TGC CC-3′) in Fig. 2b and FTD converted oligonucleotide for non-FTD sample (5′-AGA CAF GCC CAG ACA FGC CC-3′) in Fig. 2c. Importantly, in the conductance profiles, larger conductive signals around 0.41 of relative G coexisted with the conductivity of cytidine and around 0.23 of relative G in the thymine position of #6 and #16 (Fig. 2a). According to the conductance table (Table 2), T is 39.1 pS, FTD is 17.9 pS, and the relative conductance with G (86.7 pS) is 0.45 and 0.20, respectively. Based on the conductive level, the smaller signals were found to be FTD signals so that the conductance plots of the signals at # 6 and # 16 confirmed the signal derived from FTD. Such conductance peaks are also observed for conductance plots of all FTD-exposed strains (F1 and F5) (Fig. 2d). This indicates that FTD is incorporated into the p53-binding motif after incubation with FTD.

FTD incorporation instead of T in the binding DNA motif sequence depends on the duration of FTD exposure. Figure 2e shows the FTD incorporation rate for F1, and F5. The FTD incorporation rate was defined as (*n*_F_/(*n*_F_ + *n*_T_), where *n*_T_, and *n*_F_ are the number of signals of T and F, respectively (SI: S7 and Fig. S2). It was found that FTD incorporation rate increased from 10.3% (229/1984) for the F1 sample to 14.8% (345/1992) for the F5 sample. A similar FTD incorporation rate increase in the p53-binding motif was observed from F1 to F5 also for the HCT-116 cell line cells (Fig. 2f–h, SI: S4). In a previous study, FTD incorporation was also reported^7^. Similarly, in this study, in order to confirm FTD incorporation, we utilized mass spectroscopy for the FTD detection in our sample DNA from F5 cell lines, and HPLC for estimation of FTD incorporation rate in DNA (SI: S5). These results suggest that FTD are incorporated into sample DNA and the estimated FTD incorporation rate is found to be in the range of 5–10% of total thymine amount, which is comparable to FTD corporation rates determined here.

These results suggest that FTD gradually incorporates into the p53-binding motif so that the activity of p53 transcription factor and its target genes may be influenced under FTD exposure.

### Detection and quantification of FTD incorporation in DNA binding domain of three genes

Next, in order to investigate the relationship between FTD incorporation and their gene activity, we investigated the FTD incorporation rate for three binding regions of p53, NFKB3, and c-Myc for whole samples of DNA extracted from RKO cells. The DNA sequences of the binding consensus regions for p53, NFKB3, and c-Myc were determined using MotifMap^24^ (Table 3).

**Table 3:** DNA binding sequence of p53, NFKB3, and c-Myc as transcription factors.

Transcription factors	DNA binding sequence	Reference
p53	5′-AGACATGCCCAGACATGCCC-3′	Reference^20^
NFKB3	5′-CGGAGATTCC-3′	Motif ID: MA0107^a^
c-Myc	5′-ACCACGTGC-3′	Motif ID: M01145^a^

^a^Cited from the database site: http://motifmap.ics.uci.edu/.

The binding sequences for NFKB3 and c-Myc are determined based on the binding consensus sequence in MotifMap. FTD is potentially incorporated into the thymine position (red colored “T”). The binding sequence for p53 is the same as the target DNA in Table 1.

**Figure 3:**
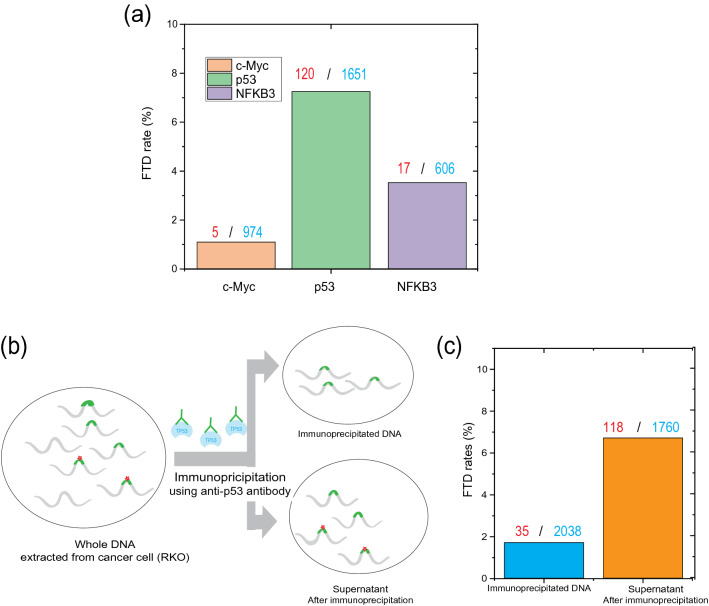
FTD incorporation of DNA and anti-p53 immunoprecipitation. (**a**) Dependency of FTD incorporation rates on the consensus-binding region of TF, i.e., c-Myc (orange), NFKB3 (purple), and p53 (green). Based on RNA analysis, the p53 and NFKB3 were impaired by FTD exposure, while c-Myc was not impaired by FTD exposure. The FTD rates for p53 and NFKB3 are larger than those for c-Myc. TRRUST, transcriptional regulatory relationships detected by sentence-based text-mining; FTD, trifluridine; TF, transcription factor. (**b**) Flowchart of the preparation of “immunoprecipitated DNA” and “supernatant DNA” by anti-p53 immunoprecipitation. To confirm the inhibition of binding ability with p53 to the binding motif, we prepared immunoprecipitated DNA using anti-p53 antibody, which was extracted from RKO cell lines, and the DNA that does not bind to anti-p53 antibody, which was the supernatant of the immunoprecipitated using anti-p53 antibody. (**c**) FTD incorporation rate for the p53 binding domain in the immunoprecipitated DNA using anti-p53 antibody (right), supernatant after immunoprecipitation using anti-p53 antibody (left).

By using the consensus sequence, the detected signals are reassembled, and FTD incorporation instead of dT in the sequence was evaluated for p53, NFKB3, and c-Myc (Fig. 3a). We found that FTD incorporation rate of p53 (7.3%: 120/1651) and NFKB3 (2.8%: 17/606) were significantly large, relative to that of c-Myc (0.5%: 5/974). On the other hand, our gene expression analysis results by microarray (SI: S8–S10) suggests the activity of p53 and NFKB3 was influenced by FTD exposure, while the activity of c-Myc was not influenced by FTD exposure. Therefore, this result suggests that FTD incorporation in the binding motif after FTD exposure influences the activity of the transcription factors in the cancer cells. These results, taken together, suggest that the DNA alteration, i.e., FTD incorporation instead of dT, at the binding motifs of p53 transcription factor could induce a change in the binding ability of p53, changing the p53-related gene expression.

### Alteration of DNA-transcription factor binding by FTD incorporation

Finally, in order to detect DNA alternation of the transcription factor binding motif and the activity of the transcription factors, we investigated the binding ability with p53 and FTD-incorporated sequence of the p53 binding motif (SI: S6). Since DNA mutations and methylation in the DNA sequence to which transcription factors bind are able to inhibit the binding of transcription factors to DNA^4,5^, it is assumed that the DNA alteration, i.e.*,* FTD incorporation, induced inhibition of the binding ability of p53 to the binding motif.

To investigate the relationship of p53’s binding ability to its binding motif due to FTD incorporation, we immunoprecipitated p53-associated DNA using anti-p53 antibody from RKO cell line cells and analyzed the anti-p53 antibody unbound DNA fraction (the supernatant) (Fig. 3b). For these two samples, we performed single-molecule detection and evaluated the FTD incorporation rate. It is expected that due to the inhibitory effect of FTD incorporation on p53 binding, FTD incorporation rate would be observed as significantly less in the unbound (supernatant) fraction than in the anti-p53 antibody-bound fraction. Figure 3c shows FTD incorporation rate for the immunoprecipitated DNA using anti-p53 antibody and unbound fraction. The FTD incorporation rate for the immunoprecipitated DNA (1.7: 35/2038) was significantly less than that for the unbound fraction (6.7%: 118/1760), which is comparable to the FTD incorporation rate of p53 binding regions (7.3%: 120/1651) in general, as was shown in Fig. 3a. This strongly suggested that the binding ability of p53 and its binding motifs could be impaired by FTD incorporation in these motifs.

In this study, we investigated the possible effects when an exogenous substance is inserted into DNA and changes the DNA sequence. For this purpose, FTD was used as a representative model of the exogenous substance inserted into DNA. Until now, there are a few studies on the effect of FTD induced DNA alternation on some of transcription factors, while there are several reports on the FTD metabolism after uptake by the body ^25–27^. Therefore, the present results demonstrate the FTD incorporation and suggest that FTD incorporation DNA alternation are closely related to the activity on the transcription factor and drug resistance, so that this methodology would be applicable not only for understanding of regulation of transcriptional activity by exogenous substances and a mechanism of action of anticancer drugs, but also as a method of molecular biological control of cells.

Overall, this study shows that FTD as a DNA disruptor can alter the properties of cells by incorporating into promoter regions in DNA, instead of T and affecting the activity of transcription factors. Therefore, this single-molecule detection of DNA sequence alteration is a useful novel methodology for understanding DNA sequence alterations related to transcription factors. This suggests that our method can directly link DNA sequence mutations caused by disruptors to the genome, such as nucleic acid analogs, to the activity of transcription factors, which may lead to the discovery of new therapeutic methods/toxic manifestations of DNA sequence/structure changes and the development of methods to control cell functions.

## Methods

### Design of artificial oligo-nucleotides

From the previous reports^23^, the binding consensus region of p53 is known to be 5′-AXX CAT GCC CAX XCA TGC CC-3′ (X = A or G). Given that, of 288 sequence samples, 236 (82%), 27 (9%) and 25 (9%) have 0, 1, and more spacers^23^, the capture oligos with 0 or 1 spacers can cover at least 91%. Although there are several kinds of potential “spacer “, *i.g.,* abasic site (natural aldehydic type and tetrahydrofuran type), inosine and so on, we utilized tetrahydrofuran-type abasic sites (1′,2′-dideoxyribose), which is called as “dSpacer”, for the spacer of capture sequence (5′-GG GCs TGY YTG GGC sTG YYT-3′: s = dSpacer) because it has any specific selectivity for any nucleotide types. The complementary probes against the p53 binding region were synthesized with the spaces for T to FTD conversion, allowing the capture and purification of the DNA with the p53 binding region in RKO and HCT-116 cells (Table 1).

### Cell culture

RKO and HCT-116 cells were purchased from the American Type Culture Collection (Manassas, VA). These cells were cultured in Dulbecco’s modified Eagle’s medium (Nacalai Tesque Inc., Kyoto, Japan) supplemented with 10% fetal bovine serum (Thermo Fisher Scientific, MA) at 37 °C in a humidified atmosphere with 5% CO_2_. RKO and HCT-116 cell line cells were grown by passaging twice a week with trypsin–EDTA and were cultured in the medium containing FTD of escalating concentrations, starting with 1 μM and final concentration is 400 μM, for about 1 month or 5 months. Mycoplasma testing was performed using the MycoAlert Mycoplasma Detection Kit (Lonza; catalog code: LT07-218, Tokyo, Japan). Mycoplasma testing confirmed negative results. Previously we reported a microarray analysis of HCT-116 cells and F5 cells^30^. This data is registered in the GEO dataset at NCBI (GSE96787). The data shows that the RNA expression levels are highly co-related between HCT-116 cells and F5 cells.

### Microarray

The microarrays were performed on cells before FTD exposure and on cells exposed for 5 months (F5), details of which were previously described^30^. The raw data are available on the Gene Expression Omnibus Website (http://www.ncbi.nlm.nih.gov/geo/) with the SuperSeries accession number (GSE96785).

### DNA extraction and preparation

Before DNA extraction, cells were washed for three times by suspending in 50 ml of phosphate-buffered saline (pH 7.4) (Sigma-Aldrich, Tokyo, Japan) and centrifuge for 5 min. DNA was isolated from cell pellets (~ 50 micro litter) of HCT-116, RKO, HCT-116 (F5), and RKO (F5) using QIAamp DNA mini kit (Qiagen, Hilden, Germany), following the manufacturer’s instructions. For the enzyme digestion, nucleotide samples were incubated with nuclease P1 (M0660S, New England Lab, Tokyo, Japan) in the condition of 10 units per 1 µg at 37 °C for 30 min, and for HPLC, followed by the incubation with alkaline phosphatase (2120A, E. coli C75, Takara, Kyoto, Japan) in the condition of 5 units per- 1 to 20 pmol nucleotide fragments at 37 °C for 30 min. To remove enzymes from digested samples, chloroform treatment and ethanol precipitation were performed.

### Gene expression analysis

To evaluate the effects of FTD exposure on transcription factors and cellular functions, transcription factor analysis and GO analysis were performed using count data of each gene in the microarrays^30^. Transcription factor analysis was performed using a list of genes whose expression was increased or decreased by more than 1.5-fold upon FTD exposure in each cell line using TRRUST v2 in Metascape^31,32^. GO analysis was performed for each cluster after clustering with iDEP.91^33^ using all gene expression data. Statistically significant (p < 0.05) gene-sets were listed.

### Fabrication of a device for sample DNA detection

The nanogap electrodes were constructed from nanofabricated mechanically controllable break junctions (MCBJs). The fabrication procedures of the MCBJs were detailed elsewhere^34,35^. A nanochannel-integrated nanogap device was utilized. A cover made of polydimethylsiloxane (PDMS; Toray Dow Corning, Tokyo, Japan) is fused with the device substrate. The PDMS cover has an in-advance microchannel that connects the hole for introducing the sample solution and the sensor’s nanochannel. PDMS is purchased from the electrophoresis electrodes are prepared by electrochemical oxidation of silver wires (Nilaco Co. Ltd, Tokyo, Japna). The oxidation of the silver wire electrode is performed in 1 M NaCl using Electrochemical Analyzer Model 1030 (ALS Co., Ltd).

### Test procedure

The sample concentration was set to be 0.10 μM. The current across the electrodes was amplified by a custom-built logarithmic current amplifier and recorded at 10 kHz and 100 kHz using a NI PXIe-4081 digital multimeter and NI PXI-5922 (National Instruments) under a DC bias voltage of 0.1 for mono-/oligo-nucleotide sample or 0.4 V for mono-nucleotide sample. After every 1 h of current–time measurement, MCBJ sample was exchanged with a new MCBJ device to keep it in a clean condition for measurements. Gap size was set to around 0.65 nm and the gap-distance was kept by feedback control of the piezo actuators.

### Immunoprecipitation

Cells were treated with formaldehyde (final concentration of 1%). Whole-cell extracts were prepared with a lysis buffer and were sonicated four times at 30-s intervals. They were then incubated with the anti-p53 antibody (sc-126; Santa Cruz Biotechnology, Santa Cruz, CA) overnight at 4 °C. Protein A&G PLUS-Agarose beads (sc-2003; Santa Cruz Biotechnology, Santa Cruz, CA) were then added, and the incubation was continued for 2 h at 4 °C. The mixture was isolated by centrifugation at 12,000 rpm for 20 s. DNA was reverse cross-linked by incubating tubes in a 67 °C water bath, mixing occasionally over two hours. DNA was extracted with phenol/chloroform/isoamyl alcohol (25:24:1).

## Supplementary Information

Supplementary Information.
