# Supplementary material for: Direct observation of DNA alterations induced by a DNA disruptor
Source: Sci Rep. 2022 Apr 28;12:6945. doi: 10.1038/s41598-022-10725-8 (PMC9050671; doi:10.1038/s41598-022-10725-8)
Supplement: Supplementary file 1 — Supplementary Information. [file 41598_2022_10725_MOESM1_ESM.docx]

Supporting Information

**Direct observation of DNA alterations induced by a DNA disruptor**

*Takahito Ohshiro^1^, Ayumu Asai,^2,#^, Masamitsu Konno^3,4^, Mayuka Ohkawa^4^, Yuki Komoto^1,2^, Ken Ofusa^3,5^, Hideshi Ishii^3,*^, Masateru Taniguchi^1*^*

**Affiliations**

1. SANKEN (The Institute of Scientific and Industrial Research), Osaka University, 8-1 Mihogaoka, Ibaraki, Osaka 567-0047, Japan.
2. Artificial Intelligence Research Center, SANKEN (The Institute of Scientific and Industrial Research) Osaka University, 8-1 Mihogaoka, Ibaraki, Osaka 567-0047, Japan.
3. Center of Medical Innovation and Translation Research, Graduate School of Medicine, Osaka University, 2-2 Yamadaoka, Suita, Osaka 560-0085, Japan.
4. Division of Tumor Biology, Research Institute for Biomedical Sciences (RIBS), Tokyo University of Science, 2641 Yamazaki, Noda, Chiba 278-8510, Japan.
5. Prophoenix Division, Food and Life-Science Laboratory, Idea Consultants, Inc., 1-24-22 Nanko-kita, Suminoe-ku, Osaka-city, Osaka 559-8519, Japan.

*To whom co-correspondences should be addressed:

Center of Medical Innovation and Translational Research, Graduate School of Medicine, Osaka University, 2-2 Yamadaoka, Suita, Osaka 565-0871, Japan.

Tel: +81-(0)6-6210-8406

Fax: +81-(0)6-6210-8407

e-mail: [hishii@gesurg.med.osaka-u.ac.jp](mailto:hishii@gesurg.med.osaka-u.ac.jp)

SANKEN (The Institute of Scientific and Industrial Research), Osaka University, 8-1 Mihogaoka, Ibaraki, Osaka 567-0047, Japan.

Tel: +81-(0)6-6879-8446

Fax: +81-(0)6-6875-2440

e-mail: [taniguti@sanken.osaka-u.ac.jp](mailto:taniguti@sanken.osaka-u.ac.jp)

*^#^The present address: xxxxx, Japan.*

Table of Contents

***S1 Single -molecule detection for FTD and FTD incorporated nucleotides***

***S2. Signal Picking and Base-calling***

***S3. Signal Assembly and Conductance Plotting***

***S4. Device repeatability and reproducibility for single-molecule electrical detection***

***S5. Detection of FTD incorporation by mass-spectroscopy and HPLC***

***S6. Capturing the target DNA including FTD incorporated DNA and non FTD incorporated DNA.***

***S7. Sensitivity and specificity for FTD and T in single-molecule detection***

***S8.  Gene expression profile of the colon cancer cell lines exposed to FTD***

***S9. Analysis of Transcriptional activity and cellular functions***

***S10. Analysis of the suppression by FTD of apoptosis and cell death.***

**S1 Single -molecule detection for FTD and FTD incorporated nucleotides.**

    We measured the nucleotide of FTD, and thymine and sample oligonucleotide extracted RKO F5 strain by using this single-molecule electrical detection and obtained the following conductance-time profiles (**Fig. S1**).


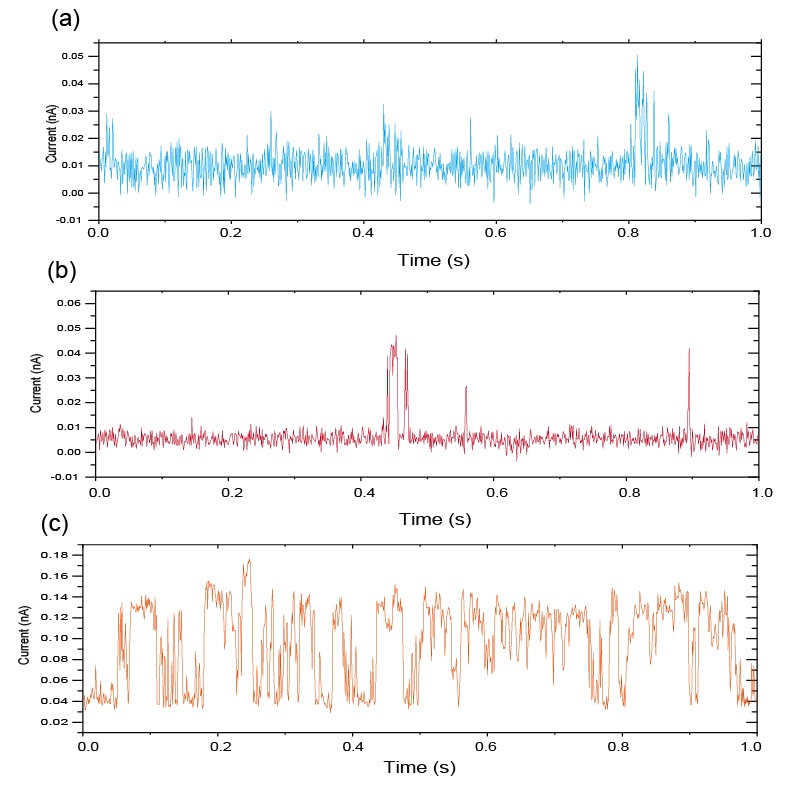


**Figure S1.** **Mono-nucleotide (FTD and T) and oligonucleotide signal detection**. (a) Typical *I*-*t* profiles for FTD (a) and T (b) in aqueous solution. (c) Typical *I*-*t* profiles for  samples, oligonucleotide extracted RKO F5 strain. The signals contain the target DNA sequence of 5'-AGA CAT GCC CAG ACA TGC CC-3’) and FTD converted oligonucleotide (5'-AGA CAF GCC CAG ACA FGC CC-3’, 5'-AGA CAT GCC CAG ACA FGC CC-3’, and so on).

**S2. Picking and base-calling.**

　    First of all, signal regions are extracted from the conductance-time profiles before signal analysis. In order to determine signal regions, the baseline level, noise-level, signal-rise time and signal-end time, should be defined.  In this study, the baseline level is defined as the mode of the histogram of the last two-thousands data points at each time, and the noise level is defined as the mode of the standard deviation of two-hundreds data points obtained by dividing the latest ten thousands data points at each time into five-hundreds equal parts. Next, based on the determined baseline, the signal-rise time is defined as the start-time of the signal from the baseline with a value exceeding six times the noise level as the threshold value, and the signal-end judgment is defined as the end-time when a data point below one time the noise level appears from the estimated baseline after the signal starts.

        The base-calling of a Phred method, which is widely used for a conventional genome sequencer analysis, is applied for this signal analysis of oligonucleotide samples. The details are previously reported **[16, 18]**. In this study, we simply show the procedures in the following. In the first step, the data-histogram was formed from the local regions of the conductance-time profiles, each of the peak values of the conductance are determined. Among the several peak values, the minimum and maximum values of the peaks are found to be corresponding to baseline, and G, respectively on the relative conductance values (**Table 1**). In the second step, by using these peak conductance and the standard deviation values, each base-probability of each base-element can be calculated.  In this calculation, we assumed each of the conductance histograms followed normal distribution so that the calculations are performed by Gaussian equation.  In the third step, base-species in the time-profiles was sequentially assigned, based on the maximum values of the probability among each of the base-type or baseline. The maximum value among each of the base-probability values was sequentially assigned to the base-type or baseline for each of the time regions. For each of the assigned bases, the accuracy of the base-assignment was quantified by the Q score. The signals with low Q values are due to the large conductance distributions and overlap of conductance distributions. In order to secure the accuracy of read-signal sequence, it is necessary to use read sequence signals with over six Q scores (P > 75%).

**S3. Signal assembly and conductance plotting**

The signal assembly was performed based on tunnel-current intensity and the detailed procedure is shown in previous reports **[17]** and briefly described in the following procedure. The sequence position number was assigned for each read-base in the “long-read signals”, compared to the reference sample sequence such as p53 binding domain (5’- AGA CAT GCC CAG ACA TGC CC -3’), where over six right-read base signals are described as “a long-right read signal” and used for assembly and plotting process. The read direction (5’ to 3’, or 3’ to 5’) was found to be sometimes changed so that the “duplicated” read signals are observed. In this signal assembly process, these duplicated-reads regions in the read sequence were automatically deleted after the sequence-position assignment and then the straight-right read region signals were used. In the assembly, we used the reference sequence (5’- AGA CAT GCC CAG ACA TGC CC -3’) and its potential FTD incorporated sequences (5’- AGA CAF GCC CAG ACA TGC CC -3’, and 5’- AGA CAT GCC CAG ACA FGC CC -3’). From the conductance-profiles of the long-right read signals, each of the conductance values at each of the base positions were plotted as shown in **Fig 2 a-c**. The signal count of FTD incorporated and non-FTD incorporated base (T) for each of base-positions are performed as the following. For instance, in the case of the #6 positions in the reference sequence, the determination of T or F in the time region are performed by the previous base-calling process (**SI: S2**). Since T and F are relatively similar relative conductance values, the selectivity and base-call accuracy is important for the signal count. The detail selectivity and accuracy for T and F are described in the next section (**SI: S7**).  Since the false F/T judgments are occurred, the false rate is subtracted from each of the signal counts for P, F1, and F5, and then F (*n*_F_) and T (*n*_T_) counts are determined. The incorporation rate of FTD was defined as (*n*_F_ / (*n*_F_ + *n*_T_) , where *n*_T_ , and *n*_F_ are the number of signals of T and F, respectively.  The FTD incorporation rate for F1 strain was found to be 10.3 % (229/1984). Similarly, The FTD incorporation rate for F5 strain was found to be 14.8 % (345/1992).

**S4. Device repeatability and reproducibility for single-molecule electrical detection**

We utilized four devices for each DNA sample extracted from the sample cell line cells, and detected the DNA signals. From all the obtained signals, we determined that the FTD incorporation rate for F1 and F5 RKO cell lines were found to be 10.3% (229/2213) 14.8% (345/2337), respectively (**Fig. 2g**) and FTD incorporation rate for F1 and F5 HCT-116 cell lines were found to be 14.0 % (715/5128) and 19.5 % (345/2337), respectively (**Fig. 2h**). In order to clarify the reproducibility by nanogap device, we show the obtained FTD incorporated signal number, non-FTD incorporation signals number, and the FTD incorporation rate for each of the devices in Table (**Tables S1** and **S2**). For RKO cell lines, the determined FTD incorporation rate for F1 and F5 was found to be in the range of 5.1 % - 14.1 %, and 11. 1% - 18.4 %, respectively (Table S1), and the standard deviation values of FTD incorporation rate for F1 and F4 were found to be 3.8 % and 3.2 %, respectively. Since the p-value was found to be 0.02 between F1 group and F4 groups, the increments of FTD incorporation rate between F1 and F4 cell lines are clear. Similarly, for HCT cell line cells, the determined FTD incorporation rate for F1 and F5 was found to be in the range of 12. % - 16.3 %, and 17. 7% - 20.8 %, respectively (Table S2), and the standard deviation values of F-rate for F1 and F4 were found to be 1.8 % and 1.4 %, respectively. Since the p-value was found to be 0.028 between F1 group and F4 groups, the increments of FTD incorporation rate between F1 and F4 cell lines are also clear.

**Table S1.** **FTD incorporation rate of sample DNA in RKO cell line cells for each nanogap-device**.

| Experiment # | Detected signal | No inserted signal | F inserted signal | F rate |
| --- | --- | --- | --- | --- |
| RKO-F1 #1 | 311 | 284 | 27 | 8.68 |
| RKO-F1 #2 | 256 | 220 | 36 | 14.1 |
| RKO-F1 #3 | 1257 | 1111 | 146 | 11.6 |
| RKO-F1 #4 | 389 | 369 | 20 | 5.14 |
| RKO-F4 #1 | 757 | 673 | 84 | 11.1 |
| RKO-F4 #2 | 436 | 361 | 75 | 17.2 |
| RKO-F4 #3 | 511 | 417 | 94 | 18.4 |
| RKO-F4 #4 | 633 | 633 | 92 | 14.5 |
| **RKO-F1 Total (#1 to #4)** | **2213** | **1984** | **229** | **10.3** |
| **RKO-F4 Total (#1 to #4)** | **2337** | **1992** | **345** | **14.8** |

We utilized ten nano-gap devices (Device: #1 to #10). For each of the devices, we performed the formation of nanogaps for every single-molecule electrical measurement. In order to demonstrate the repeatability of the gap formation, we showed the in Table S2. We confirmed the nanogap formation per one device at least 20 times and at least fifty experimental runs, while the signal detection time is five minutes per one run. By using these devices, the averaged measured time length for each of the devices was found to be at least than four hours as shown in the column of ‘Ex time’ in Table S2.

**Table S2.** **Repeatability for each nanogap-device**.

| Device Number | Gap formation (n) | Ex Run (n) | Ex time (h) |
| --- | --- | --- | --- |
| #1 | 34 | 240 | 20 |
| #2 | 34 | 144 | 12 |
| #3 | 48 | 58 | 4.8 |
| #4 | 23 | 480 | 40 |
| #5 | 24 | 1442 | 120 |
| #6 | 24 | 1032 | 86 |
| #7 | 36 | 225 | 18.8 |
| #8 | 55 | 602 | 50 |
| #9 | 29 | 1224 | 102 |
| #10 | 30 | 602 | 50.2 |

**S5. Detection of FTD incorporation by mass-spectroscopy and HPLC**

In order to investigate the FTD incorporation into sample DNA extracted from cancer cells after FTD exposure, the presence of FTDs in DNA extracted from HCT-116 (F5) cancer cells are investigated by the results of ESI MS-spectroscopy. In these experiments, the sample DNA from cancer cells before FTD exposure of HCT-116 (P) and after FTD exposure (F5), were extracted, and then the extracted DNA was hydrolyzed by DNAase, and then these sample DNA were measured by ion-trap mass spectrometer (LTQ Orbitrap XL™, Thermo Fisher Scientific, USA) with negative mode. From the mass spectrum for DNA extracted from P and F5 cells (**Fig.S2 a-d**), the FTD peak (C_10_H_11_F_3_N_2_O_8_P^-^, expected m/z: 375.17) was detected for DNA extracted from F5 cells (m/z: 375.5143, intensity 3000) (**Fig. S2a**), while the FTD peak was not detected for DNA extracted from P cells (no peak from m/z: 373-376) (**Fig. S2b**). On the other hand, the thymidine peak (C_10_H_14_N_2_O_8_P^-^, expected m/z: 321.049) was detected for both sample DNA extracted from F5 cell (m/z: 321.06) (**Fig. S2c**) and P cells (m/z: 321.0491) (**Fig. S2d**). Therefore, this spectrum result supports the experimental results of the detection of FTDs by the nanogap electrode.

*
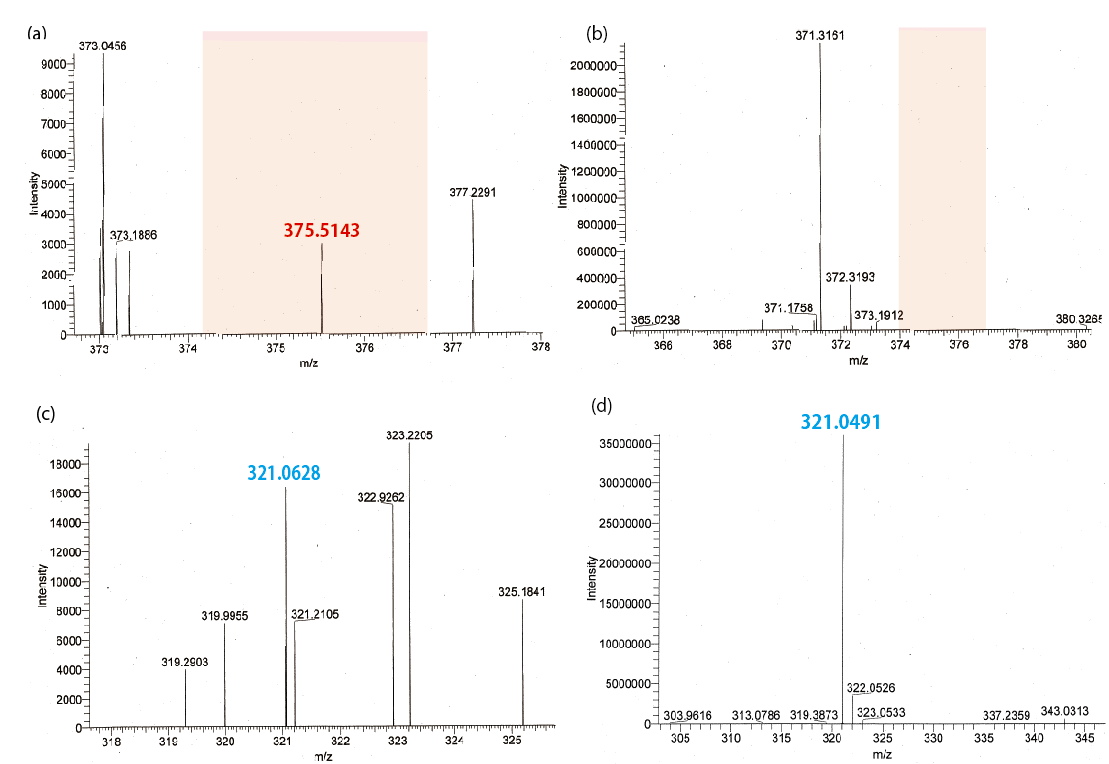
*

**Figure S2**. Mass spectrum by ESI MS-spectroscopy for DNA extracted from cancer cells after five months FTD exposure (F5) cells for (a) and before FTD exposure cells (P) for (b). The ion-trap mass spectrometer (LTQ Orbitrap XL™, Thermo Fisher Scientific, USA) are used with negative mode. The FTD peak (C_10_H_11_F_3_N_2_O_8_P^-^, expected m/z: 375.17) was found only for (a), not for (b). The red region of 374 to 376.5 (m/z) are colored for eye guide. Mass spectrum by ESI MS-spectroscopy for DNA extracted from cancer cells before FTD exposure after five months FTD exposure (F5) cells for (c) and before FTD exposure cells (P) for (d). The FTD peak (C_10_H_14_N_2_O_8_P^-^, expected m/z: 321.049) was found both for (c) (m/z: 321.06) and for (d) (m/z: 321.05).

Next, in order to estimate the FTD incorporation rate into sample DNA, we utilized HPLC for whole sample DNA extracted from HCT-116 (F5) cell lines. For the HPLC experiment, we performed as follows: the monitoring wavelength, 254 nm; mobile phase, 2% acetonitrile and 0.01% trifluoroacetic acid; column, Agilent Poroshell 120, EC-C18, 3.0 i.d. x 150 mm, 2.7 mm (EC-C18: End Capped C18); apply volume, 50 mL; flow rate, 0.5 mL/min, in the HPLC equipment (Agilent HPLC System 1100, Agilent, Tokyo, Japan). For the experimental control, nucleoside, analytical standard, Supelco (47310-U, Sigma-Aldrich, Tokyo, Japan), Trifluorothymidine (T2511, Tokyo Chemical Industry, Tokyo Japan) were used. Based on the dT and FTD peak integration in the HPLC, the FTD per dT for HCT and RKO were found to be 5.9 % and 5.7 % respectively so that the FTD incorporation rate is estimated as in the range of several percent of the total thymine amount.

In addition, compared the signal intensities of the thymine (C_10_H_14_N_2_O_8_P^-^, expected m/z: 321.05) to that of FTD (C_10_H_11_F_3_N_2_O_8_P^-^, expected m/z: 375.17) in mass-spectrum for the sample DNA (F5 cancer cell lines), the peak intensity of thymine (m/z: 321.06, intensity: 16,000) was fifth times larger than that of FTD (m/z: 375.5143, intensity: 3000). This suggests that FTD molecules would be incorporated into around ten percent of all the thymine positions instead of thymine. Therefore, these results suggested that the FTD incorporation rate determined by our single-molecule electrical method by nanogap device is in the range of reasonable FTD incorporation rate values.

**S6. Capturing the target DNA including FTD incorporated DNA and non FTD incorporated DNA.**

In this study, the sequence (5’-GG GCs TGY YTG GGC sTG YYT- 3’) is used for capturing the target p53 binding DNA domain. Since the space (s), *i.e.,* abasic site, has no selectivity for both of the potential target, *i.e.,* thymine (T) and its fluorinated thymine (FTD: trifluridine), the designed sequence would capture the FTD incorporated DNA and non-FTD incorporated DNA. In order to experimentally investigate the difference in the efficiency of capture between FTD-incorporated DNA vs non FTD-incorporated DNA, the captured efficiency of DNA extracted probe DNA between P cell (before FTD exposure) and F5 (after FTD exposure) are shown in **Table S3**. Based on the results, there is no significant difference in the capture DNA efficiency between P cell (before FTD exposure) and F5 (after FTD exposure) as shown in Table S. This DNA capture efficiency data suggest our designed probe DNA worked well as expected.

**Table S3: Comparison of captured efficiency of DNA volume between P and F5.**

| Experiment # | P cell: Total captured (μg/0.1mL) | F5 cell: Total captured (μg/0.1mL) | F5 cell/ P cell |
| --- | --- | --- | --- |
| #1 DLD | 29.26 | 26.83 | 0.92 |
| #2 DLD | 69.51 | 45.45 | 0.65 |
| #3 DLD | 27.40 | 29.31 | 1.07 |
| #1 RKO | 27.91 | 22.38 | 0.80 |
| #2 RKO | 30.29 | 30.87 | 1.02 |

**S7.  Sensitivity and specificity for FTD and T in single-molecule detection**

       In order to detect the FTD incorporation instead of T, we statistically compared FTD signal with thymine (T). From signal conductance-time profiles, several kinds of the feature signal values, the maximum value of the conductance, the duration of the signal, and the standard deviation of the conductance, and signal shape factors, were obtained. The detailed estimation procedure of the signal-shape factors is shown in the next section. These signal feature values reflected the difference between FTD and T. For instance, the histogram of the maximum value of the conductance profile demonstrated the characteristic conductance values for each of the nucleotides **[17]**. From the previous study, it was found that the difference in the maximum value of the conductance profile is closely related to the HOMO level of the nucleotide molecules **[16, 17]**. In this two-class problem (FTD or T), we can assign the signals obtained in FTD nucleotide solutions as “true FTD signals” and the signals obtained in the T solutions as “true non-FTD signals. On the other hand, by using the classifier as mentioned above, we can predict the ‘predicted’ FTD signals or ‘predicted’ non-FTD signals for test signals. Therefore, all the test signals were categorized to four groups; ‘correctly predicted and true FTD (true positive)’, ‘correctly predicted and true no-FTD (true negative)’, ‘predicted FTD but true non-FTD (false negative)’, ‘predicted non-FTD but true FTD (false positive)’, where “true signals” means correctly predicted methylated nucleotide species, “false signals” means incorrectly predicted event values. These four values are summarized as a confusion matrix (**Fig.S3a**). In this study, the accuracy of FTD/non-FTD judgments by the cross-validation of the test data are shown in a confusion matrix of **Fig. S3a**. From these classification metrics such as precision, recall, specificity and sensitivity of our classifier, the F-measure for FTD (F) and non-FTD (T) of thymine was found to be 0.75. Based on the training results, we determined the mixing ratio of FTD and non-methylated adenosine (A). We prepared the ratio of F molecule number to total number of molecules counted; 5%, 20%, 40%, 60%, 80%, and 95%. The real F ratio is very similar to the determined F ratio (**Fig. S3b**). This indicates that it is also possible to identify methylated mixture in the RNA sequence.


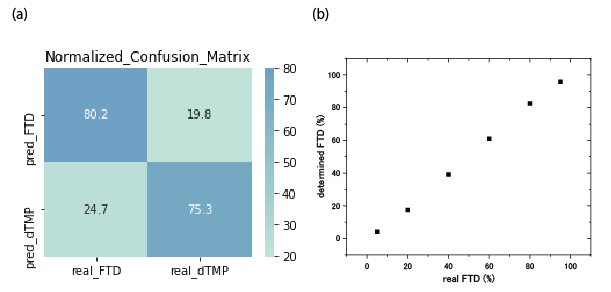


**Figure S3 (a) Confusion matrix for F and T.** There are four groups of signal counts; ‘correctly predicted and true FTD (true positive: left upper)’, ‘correctly predicted and true no-FTD (true negative: right upper)’, ‘predicted FTD but true non-FTD (false negative: left lower)’, ‘predicted non-FTD but true FTD (false positive: right lower)’, where “true signals” means correctly predicted methylated nucleotide species, “false signals” means incorrectly predicted event values. **(b) Comparison of the original sample concentration ratio and the determined concentration ratio for FTD/T mixed signals.** We prepared the ratio of F molecule number to total number of molecules counted; 5%, 20%, 40%, 60%, 80%, and 95%. The real F ratio is very similar to the determined F ratio.

**S8.  Gene expression profile of the colon cancer cell lines exposed to FTD**

We investigated whether the p53 target genes were affected by the DNA mutation in the binding region of p53 by FTD exposure, we performed gene expression analysis for each sample cell line (RKO and HCT-116) cells using mRNA microarrays. This makes it possible to identify transcription factors including p53 and cellular functions that are affected by FTD exposure. Total RNA was extracted from cells before and after exposure to FTD, and transcriptional regulatory relationships detected by sentence-based text-mining (TRRUST) analysis, and gene ontology (GO) analyses were performed using results of the gene expression analysis with microarrays.

In the TRRUST analysis, transcription factors impaired by FTD exposure were evaluated using each list of genes whose gene expression was decreased by more than 1.5-fold in RKO cells and HCT-116 cells by FTD exposure. As the results, the activity of 43 and 57 transcription factors was impaired in RKO and HCT-116 cells, respectively, by FTD exposure, and 20 transcription factors with particularly impaired activity were identified (**SI: S9**). After omitting the mutated transcription factors, four transcription factors (*TP53*, *RELA* (encoding NFKB3), *VHL* (encoding Vhl), and *HIF1A* (encoding HIF1-alpha)) were impaired in the transcription function by FTD exposure **(Fig. S4 and S5)**. GO analysis also confirmed that apoptosis and cell death were decreased by FTD exposure **(Fig. S6)**. It has been reported that FTD exposure activates the p53 pathway and induces apoptosis^21, 22^. In addition, these apoptosis and cell death are also detected by our studied living cell cells by counting with trypan blue exclusion methods (**SI:S9**). In this study, the cells were exposed to FTD for a long period so that FTD can be detected in the DNA. The result of this study in which cell death and apoptosis may be suppressed by impairing the p53 pathway. Therefore, these results suggest that FTD incorporation in the p53-binding motifs after FTD exposure may be closely related to the change in the transcriptional activity of p53 related apoptosis in cancer cells.


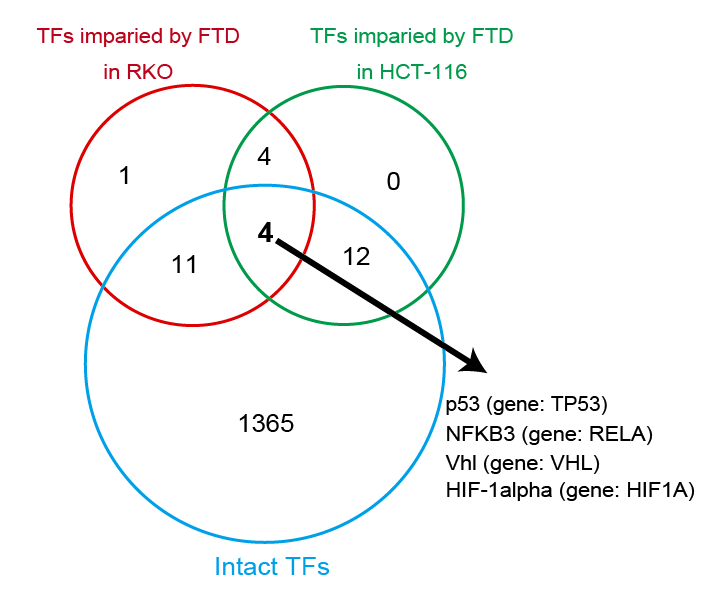


**Figure S4** Clusters of genes influenced by FTD exposure in RKO and HCT-116 cells based on List of the top 20 TF activated by FTD exposure as determined by TRRUST analysis. The red, green, sky blue circle represents the TF clusters by impaired FTD in RKO cell, in HCT-116 cell, and intact TFs, respectively.

**S9. Analysis of Transcriptional activity and cellular functions.**

In the TRRUST analysis, transcription factors impaired by FTD exposure were evaluated using each list of genes whose gene expression was decreased by more than 1.5-fold in RKO cells and HCT-116 by FTD exposure. From the results, the activity of 43 and 57 transcription factors was impaired in RKO and HCT-116 cells, respectively. **Fig.S5 a and b** show the top 20 genes list for RKO and HCT-116, respectively. Among these genes in the list, there are some transcription factors that are not intact (**Table S5**) so that the intact genes are found to be RELA, and TP53. Gene ontology analysis shows cellular functions affected by FTD exposure. Fig.S3 shows the cellular functions such as apoptosis and cell death were decreased by FTD exposure.

**
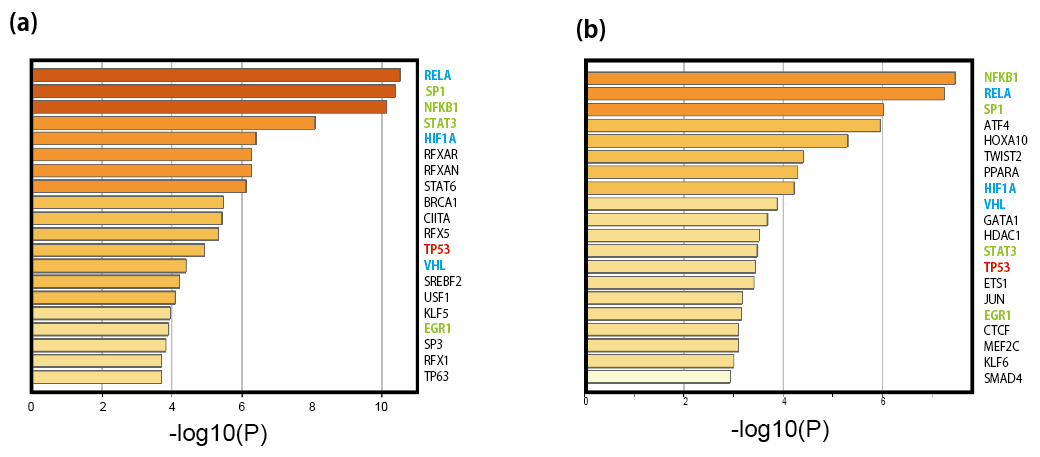
**

**Figure S5. FTD affects transcriptional activity and cellular functions.** (a, b) List of the top 20 transcription factors particularly impaired by FTD exposure as determined by TRRUST analysis: Left; (a) HCT-116 cell, Right; (b) RKO cell. The sky-blue (RELA, HF1A, VHL) and red (TP53) colored transcription factors are listed in the intact ones in Table S1. On the other hand, yellow-green colored transcription factors are not listed in Table S1.


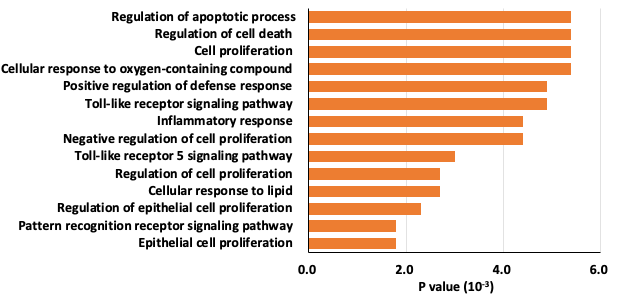


**Figure S6. Cellular functions altered by FTD exposure.** List of cellular functions impaired by FTD exposure. The list was determined by GO analysis using a list of genes whose gene expression was commonly decreased in RKO and HCT-116 cells by FTD exposure.

**S10. Analysis of the suppression by FTD of apoptosis and cell death**

In order to confirm the suppression by FTD of the apoptosis and cell death, we performed the cell death study for the HCT cells before and after FTD exposure. In this experiment, we studied living cell cells by counting with trypan blue exclusion methods to discriminate between variable cells and dead cells (**Table S4**). The results indicated that the parent HCT116 cells showed more than 96% viability, whereas the exposure of HCT116 cells to FTD in culture resulted in 71% viability, suggesting that 25% of parent cells executed the cell death in the experiment. This result indicates direct evidence for the effect of acute exposure to FTD.

**Table S4. Cell death study for HCT and RKO cell before and after FTD exposure**

| Experiment # | Total (×10^6^ /ml) | Live (×10^6^ /ml) | Dead (×10^6^ /ml) | Viability (%) |
| --- | --- | --- | --- | --- |
| HCT-P #1 | 2.4 | 2.3 | 0.1 | 96 |
| HCT-P #2 | 2.4 | 2.3 | 0.1 | 97 |
| HCT-F5 #1 | 1.1 | 0.78 | 3.3 | 70 |
| HCT-F5 #2 | 1.1 | 0.71 | 3.9 | 64 |
| HCT-F5 #3 | 1.1 | 0.89 | 3.2 | 74 |
| HCT-F5 #4 | 1.1 | 0.88 | 2.6 | 77 |

**Table S5. Intact transcription factors in RKO cells and HCT-116 cells.** This list was created by integrating the list of 1988 human transcription factors compiled in a previous study **[30]** and the mutation information of RKO and HCT-116 cells in [Genomics of Drug Sensitivity in Cancer](https://www.cancerrxgene.org/) (https://www.cancerrxgene.org).

| **AES** | **NFE2L1** | **ZNF143** | **CBX3** | **ZNF655** |
| --- | --- | --- | --- | --- |
| **NR0B1** | **NFE2L2** | **ZBTB16** | **TFEC** | **DDX54** |
| **ALX3** | **NFIB** | **ZNF146** | **ZNFN1A3** | **ZNF426** |
| **APBB1** | **NFIC** | **TRIM25** | **ZNFN1A2** | **ZSCAN5** |
| **APC** | **NFIL3** | **ZNF154** | **ATF5** | **ZNF343** |
| **PHOX2A** | **NFIX** | **ZNF155** | **MTF2** | **ZNF557** |
| **ARNT** | **NFKB2** | **ZNF161** | **RBM16** | **BHLHB3** |
| **ARNTL** | **NFKBIA** | **ZNF165** | **ZNF409** | **NSBP1** |
| **ASCL1** | **NFKBIL1** | **ZNF174** | **ZFP30** | **LASS4** |
| **ASCL2** | **NFKBIL2** | **ZNF177** | **KIAA0863** | **HMBOX1** |
| **ATBF1** | **NFX1** | **ZNF180** | **ZNF510** | **FLJ11795** |
| **ATF1** | **NFYA** | **ZNF184** | **MLXIP** | **FLJ23436** |
| **ATF3** | **NFYB** | **ZNF187** | **ANKRD6** | **LIN28** |
| **ATF4** | **NHLH1** | **ZNF189** | **FOXJ3** | **E2F8** |
| **ATOH1** | **NHLH2** | **ZNF192** | **ZBTB1** | **SNIP1** |
| **BAPX1** | **NKX3-1** | **ZNF193** | **HABP4** | **ZNF408** |
| **BARD1** | **NME2** | **ZNF195** | **SIRT2** | **ALS2CR8** |
| **BCL3** | **NONO** | **ZNF198** | **ELL2** | **ZBTB3** |
| **BCL6** | **CNOT3** | **ZNF200** | **SNW1** | **ZNF669** |
| **PCGF4** | **NOTCH4** | **ZNF202** | **DUX4** | **HDAC11** |
| **POLR3D** | **NPM1** | **ZNF205** | **TRIM32** | **ZNF671** |
| **FOXL2** | **NRL** | **ZNF207** | **SCMH1** | **ZNF613** |
| **ZFP36L2** | **YBX1** | **ZNF213** | **DIP2C** | **NANOG** |
| **KLF9** | **NR4A2** | **ZNF214** | **FBXL11** | **PHF17** |
| **BTF3** | **SIX6** | **ZNF223** | **KIAA0194** | **ZNF442** |
| **BTG1** | **ORC2L** | **ZNF224** | **SPEN** | **ANKRD53** |
| **RUNX2** | **OTX1** | **ZNF225** | **JMJD2B** | **ZNF606** |
| **CBFA2T3** | **OTX2** | **ZNF226** | **ZNF292** | **ZFP2** |
| **RUNX3** | **OVOL1** | **ZNF227** | **ZHX3** | **NARG1** |
| **CDC5L** | **PA2G4** | **ZNF228** | **JMJD2C** | **ELL3** |
| **CDX1** | **PARN** | **ZNF229** | **ZBTB43** | **EPC1** |
| **CEBPA** | **PAX2** | **ZNF230** | **TBC1D2B** | **ZNF306** |
| **CEBPB** | **PAX3** | **ZNF236** | **HIC2** | **ZNF435** |
| **CEBPD** | **PAX4** | **BTG2** | **C19orf7** | **ESX1** |
| **CEBPG** | **PAX5** | **PAX8** | **SIN3B** | **PBX4** |
| **CHES1** | **PAX9** | **BRPF1** | **CUTL2** | **THAP7** |
| **ERCC8** | **PBX1** | **RDBP** | **PHF15** | **ZNF34** |
| **KLF6** | **PBX2** | **TFEB** | **SIRT5** | **ZNF436** |
| **CREB1** | **PCBD1** | **MYST3** | **SIRT4** | **ZFP91** |
| **ATF2** | **PER1** | **NR4A3** | **SIRT3** | **ZNF611** |
| **CREBL1** | **PITX1** | **LHX3** | **SIRT1** | **ZNF93** |
| **CREM** | **PITX2** | **NCOA4** | **RYBP** | **MXD3** |
| **CRX** | **PITX3** | **CSRP3** | **TARDBP** | **SOX7** |
| **CRY1** | **PKNOX1** | **FOSL1** | **HEY1** | **TFAP2D** |
| **CSRP2** | **PLAG1** | **MLL2** | **CBX7** | **KLF16** |
| **NKX2-5** | **PLRG1** | **YEATS4** | **HEY2** | **MIXL1** |
| **CTNNB1** | **PMS1** | **HMGA2** | **SUZ12** | **HDAC10** |
| **CUTL1** | **PMS2L3** | **CART1** | **MYST4** | **BRIP1** |
| **DAB2** | **PRRX1** | **ZNF239** | **SEC14L2** | **PCBD2** |
| **DAXX** | **PNN** | **NCOA3** | **RBM9** | **PCGF6** |
| **DDB2** | **POLR2B** | **LZTR1** | **ZNF278** | **ZNF394** |
| **DDIT3** | **POLR2C** | **RP13-297E16.1** | **ZIM2** | **ARID5B** |
| **DDX5** | **POLR2D** | **SMCX** | **SPO11** | **ASCC2** |
| **DLX2** | **POLR2E** | **SMCY** | **SSBP2** | **SLA2** |
| **DLX4** | **POLR2F** | **EOMES** | **TRIM29** | **MAF1** |
| **DLX5** | **POLR2G** | **HIST1H2BG** | **ZFP95** | **ZDHHC16** |
| **DMRT1** | **POLR2H** | **HIST1H2BN** | **MAFF** | **PHF6** |
| **DNMT3A** | **POLR2I** | **HIST1H2BE** | **TBC1D22A** | **ZNF397** |
| **DR1** | **POLR2J** | **HIST1H2BH** | **RAD54B** | **MKI67IP** |
| **TSC22D3** | **POLR2K** | **HIST1H2BC** | **FBXO7** | **ZNF333** |
| **E2F1** | **POLR2L** | **HIST2H2BE** | **ZNF324** | **ZNF512** |
| **E2F2** | **POU1F1** | **SOX14** | **VAX2** | **L3MBTL3** |
| **E2F3** | **POU2AF1** | **NR0B2** | **CCRN4L** | **MLR2** |
| **E2F4** | **POU2F1** | **UTF1** | **POU2F3** | **NKX6-2** |
| **E2F5** | **POU2F2** | **KLF11** | **ZNF345** | **MCM8** |
| **E2F6** | **POU3F1** | **SUPT3H** | **POLR1A** | **HOP** |
| **E4F1** | **POU3F2** | **SMARCA5** | **ZNF473** | **PEPP-2** |
| **EBF** | **POU3F3** | **IKBKG** | **RNF19** | **PGBD1** |
| **EEF1A1** | **POU4F1** | **GCM1** | **COBRA1** | **ZBTB37** |
| **EGR3** | **POU5F1** | **CSDA** | **WWTR1** | **KIAA1862** |
| **EGR4** | **POU5F1P1** | **CBX4** | **ZNF385** | **SPZ1** |
| **ELF1** | **PPARD** | **BARX2** | **ANKRD25** | **ZNF347** |
| **ELF2** | **PPARG** | **PIR** | **MIZF** | **FBXL10** |
| **ELF3** | **PPARBP** | **BHLHB2** | **ZZZ3** | **CREB3L3** |
| **ELF4** | **EIF2AK2** | **RUVBL1** | **L3MBTL** | **HDGF2** |
| **ELK4** | **PROP1** | **KLF7** | **SS18L1** | **ZNF496** |
| **EMX1** | **PTMA** | **RFXANK** | **ZNF500** | **RAXL1** |
| **EMX2** | **PURA** | **TP73L** | **RAI14** | **PHF5A** |
| **EN1** | **RAD51** | **JRK** | **PYGO1** | **ZNF499** |
| **ENO1** | **RAD51L1** | **SFRS9** | **ZNF337** | **ZNF206** |
| **EPAS1** | **RARA** | **EDF1** | **GMEB2** | **ZNF382** |
| **ERCC2** | **RARG** | **C19orf2** | **MYCBP** | **ZNF587** |
| **ERCC6** | **RB1** | **EED** | **EHF** | **FLJ14768** |
| **ERF** | **JARID1A** | **CREG1** | **LHX6** | **ZNF566** |
| **ERG** | **RBBP4** | **TRIM24** | **P8** | **C20orf100** |
| **ESR1** | **RBL1** | **SAP30** | **AATF** | **TRIM5** |
| **ESRRB** | **RBL2** | **HESX1** | **DUX3** | **EAF1** |
| **ETS1** | **REL** | **HDAC3** | **HBP1** | **NKD2** |
| **ETS2** | **RELA** | **TSC22D1** | **ZNF285** | **PRIC285** |
| **ETV2** | **RELB** | **PCAF** | **ZRF1** | **SCRT2** |
| **ETV3** | **DPF2** | **PER2** | **FOXD3** | **MBD3L1** |
| **ETV4** | **RFC1** | **BUD31** | **FOXB1** | **HSFY1** |
| **ETV5** | **RFP** | **PRPF4B** | **ANKRD1** | **TRIM15** |
| **ETV6** | **RFX1** | **TIMELESS** | **FOXP1** | **LHX4** |
| **EVI1** | **RFX2** | **FOXH1** | **TAF5L** | **ZNF30** |
| **EVX1** | **RFX5** | **PHOX2B** | **ZBTB11** | **TGIF2LX** |
| **EWSR1** | **RFXAP** | **MBD2** | **BRPF3** | **LOC90321** |
| **EYA3** | **RING1** | **FUBP3** | **CSDC2** | **ZNF160** |
| **EZH1** | **RLF** | **HIST1H2BJ** | **HNRNPG-T** | **THRAP6** |
| **FALZ** | **RNF2** | **H1FX** | **ZNF544** | **ZNF700** |
| **FHL2** | **RNF4** | **LIMD1** | **RBMX** | **ZNF439** |
| **FOXG1B** | **RORB** | **TAF1B** | **TNRC9** | **ZNF486** |
| **FOXG1A** | **RORC** | **TAF1A** | **ZNF638** | **TGIF2LY** |
| **FOXG1C** | **RREB1** | **BAZ1B** | **HIPK2** | **PYGO2** |
| **FOXF2** | **RXRA** | **BTAF1** | **KLF15** | **SPOCD1** |
| **FOXC1** | **RXRB** | **AIP** | **MED4** | **LASS5** |
| **FOXD4** | **RXRG** | **PIAS2** | **SAP30BP** | **ZNF502** |
| **FOXE3** | **SAFB** | **ASH2L** | **BRD7** | **CHURC1** |
| **FOXJ1** | **SALL1** | **LDB2** | **ABT1** | **LOC91661** |
| **FOXE1** | **SATB1** | **VCY** | **ZDHHC1** | **YTHDC1** |
| **FOXD2** | **SCML1** | **TBX19** | **TFCP2L1** | **ZNF300** |
| **FKHL18** | **SFPQ** | **MTA1** | **CNOT7** | **GIOT-1** |
| **FOXO1A** | **SFRS1** | **DEDD** | **SERTAD3** | **MGC13138** |
| **FOXO3A** | **SFRS2** | **LRRFIP1** | **DNMT3L** | **ZNF276** |
| **FMR1** | **SFRS3** | **PTTG1** | **SERTAD1** | **PRDM6** |
| **FOSB** | **SFRS5** | **ZBTB22** | **LASS2** | **ZNF670** |
| **FOSL2** | **SFRS6** | **CRSP2** | **NRBF2** | **MYOCD** |
| **NR5A2** | **SFRS8** | **ZNF235** | **TBX21** | **ZNF101** |
| **NR5A1** | **SHOX1** | **KLF4** | **RAX** | **C21orf66** |
| **FUS** | **SIM1** | **COPS2** | **VSX1** | **FOXQ1** |
| **XRCC6** | **SIM2** | **TRIP13** | **KCNIP3** | **MED8** |
| **GABPB2** | **SIX1** | **HMGN3** | **CXXC1** | **ZNF257** |
| **GATA1** | **SIX3** | **ZNHIT3** | **ZNRD1** | **ZIM3** |
| **GATA2** | **SKI** | **GTF3C5** | **TAX1BP3** | **TOE1** |
| **GATA4** | **SNAI2** | **GTF3C3** | **ZNF295** | **ZNF554** |
| **GATA6** | **SMARCA1** | **CNOT8** | **NEUROG3** | **ZNF501** |
| **GBX2** | **SMARCA3** | **TCEAL1** | **IRX4** | **ZNF653** |
| **GFI1** | **SMARCB1** | **LHX2** | **RNF141** | **OLIG1** |
| **GRLF1** | **SMARCC1** | **ZNF265** | **FOXP3** | **CENTG3** |
| **GSCL** | **SMARCD1** | **SURB7** | **TBX22** | **DACH2** |
| **GTF2A1** | **SMARCD2** | **HAND1** | **MED31** | **TWIST2** |
| **GTF2A2** | **SMARCE1** | **ZNF264** | **ASCC1** | **ZNF354B** |
| **GTF2E1** | **SNAI1** | **CRSP3** | **BOLA1** | **ZNF488** |
| **GTF2F1** | **SNAPC1** | **CRSP6** | **ZNF593** | **ANKRD22** |
| **GTF2F2** | **SNAPC3** | **CRSP7** | **POLR1D** | **ZNF641** |
| **GTF2H2** | **SNRPB** | **CRSP8** | **MLXIPL** | **BTBD11** |
| **GTF2H3** | **SNRPD1** | **CRSP9** | **ZDHHC9** | **FOXN4** |
| **GTF2H4** | **SOLH** | **TRFP** | **ASB3** | **JDP2** |
| **GTF3A** | **SOX3** | **TBPL1** | **RNF12** | **ANKS3** |
| **GTF3C2** | **SOX5** | **POLR1C** | **LEF1** | **ZNF441** |
| **H1F0** | **SOX10** | **ZNF254** | **ZNF639** | **ZNF491** |
| **HIST1H1C** | **SOX11** | **SOX13** | **ZDHHC2** | **LOC126295** |
| **HIST1H1D** | **SOX15** | **RNPC2** | **ZNF219** | **DMBX1** |
| **HIST1H1E** | **SP3** | **CREB5** | **PHF20** | **C1orf83** |
| **HIST1H1B** | **SP4** | **NFE2L3** | **TFDP3** | **HMGB4** |
| **HIST1H1T** | **SP100** | **RNF14** | **ZNF571** | **ZBTB8** |
| **HIST1H2BD** | **SREBF2** | **ZNF592** | **PHF21A** | **HMG4L** |
| **HIST1H2BB** | **SRF** | **SH2BP1** | **ZBTB7A** | **SUHW1** |
| **HCFC1** | **SRY** | **ZNF432** | **LW-1** | **LOC129138** |
| **HCLS1** | **TRIM21** | **JMJD2A** | **ZNF588** | **ZNF513** |
| **HD** | **SSRP1** | **BZW1** | **VGLL1** | **JMY** |
| **HDGF** | **SSX1** | **ST18** | **PRRX2** | **FLJ25680** |
| **HIF1A** | **SSX2** | **ZNF646** | **SFMBT1** | **DKFZp762I137** |
| **HIP2** | **SSX4** | **ZNF96** | **TH1L** | **KLF14** |
| **HIVEP2** | **STAT5A** | **MLL4** | **ETV7** | **ASB10** |
| **HKR3** | **SUPT4H1** | **PHF16** | **PHF7** | **C7orf11** |
| **HLXB9** | **SUPT5H** | **DHX38** | **ZNF581** | **CXorf43** |
| **HLF** | **TADA2L** | **SERTAD2** | **HDAC7A** | **ASB11** |
| **HLX1** | **TAF4** | **DAZAP2** | **PCQAP** | **ASB6** |
| **HMGB2** | **TAF5** | **TSC22D2** | **TRIM33** | **ASB8** |
| **HMGN1** | **TAF9** | **ZNF623** | **TAF9B** | **ASB9** |
| **HMGN2** | **TAF10** | **ZFHX1B** | **KLF13** | **ZFP28** |
| **HMGA1** | **TAF11** | **ZBTB24** | **ASB1** | **GATA5** |
| **NR4A1** | **TAF12** | **C14orf92** | **ASB4** | **BTBD4** |
| **HMX1** | **TAF13** | **SUPT7L** | **SUFU** | **SUHW2** |
| **FOXA1** | **TAL2** | **ZBTB40** | **RAP80** | **C20orf151** |
| **FOXA2** | **TARBP2** | **ZBTB5** | **POLR3K** | **ASB12** |
| **FOXA3** | **TBX1** | **THRAP3** | **HBXAP** | **GSC** |
| **HNF4A** | **TBL1X** | **THRAP1** | **SIX4** | **ZFP90** |
| **HNF4G** | **TBP** | **NR1I3** | **ZDHHC13** | **ZNF597** |
| **ONECUT1** | **TBX2** | **NR1H4** | **ING3** | **FLJ32130** |
| **HNRPAB** | **TCEA1** | **DMTF1** | **SCAND2** | **ZNF688** |
| **HNRPD** | **TCEA3** | **MED6** | **HES2** | **ZNF578** |
| **HNRPK** | **TCEB1** | **NR2E3** | **HSMPP8** | **ZNF418** |
| **TLX2** | **TCEB2** | **ZBTB33** | **ZNF586** | **ZNF417** |
| **HOXA1** | **TBX3** | **HDAC5** | **GATAD2A** | **ZNF560** |
| **HOXA4** | **TCF1** | **THRAP5** | **ANKRD49** | **ZNF524** |
| **HOXA5** | **TCF2** | **HMG2L1** | **CASZ1** | **ZNF563** |
| **HOXA6** | **TCF3** | **NR1H3** | **WHSC1L1** | **SIX5** |
| **HOXA7** | **TCF7** | **PQBP1** | **ZNF434** | **ZNF420** |
| **HOXA9** | **TCF7L2** | **PREB** | **ZSCAN2** | **ZNF565** |
| **HOXA10** | **TCF8** | **ZNF263** | **ANKZF1** | **ZNF582** |
| **HOXA11** | **C2orf3** | **YAF2** | **PB1** | **LOC148203** |
| **HOXA13** | **TCF12** | **TOB1** | **PHF10** | **ZNF555** |
| **HOXB1** | **TCF15** | **SFRS14** | **ZNF444** | **ZNF570** |
| **HOXB3** | **ZNF354A** | **TRIM28** | **C14orf106** | **PHF13** |
| **HOXB4** | **TCF19** | **ZNF197** | **SNFT** | **GLIS1** |
| **HOXB5** | **MLX** | **ZNF256** | **HSZFP36** | **SMYD1** |
| **HOXB6** | **TCOF1** | **RBM7** | **ZDHHC7** | **NFXL1** |
| **HOXB7** | **TEAD1** | **THOC4** | **PNRC2** | **ZNF595** |
| **HOXB8** | **TEAD4** | **TOPORS** | **ZNF673** | **FLJ25169** |
| **HOXB9** | **TEAD3** | **SSX3** | **ZNF416** | **OTEX** |
| **HOXC4** | **TEF** | **OLIG2** | **ZNF446** | **NKX2-3** |
| **HOXC6** | **TFAP2A** | **CTDSPL** | **YEATS2** | **ZFP1** |
| **HOXC8** | **TFAP2B** | **HNRPR** | **ZNF334** | **ZNF519** |
| **HOXC10** | **TFAP4** | **SRRM1** | **DCP1A** | **ZNF610** |
| **HOXC11** | **TFCP2** | **IRX5** | **TRERF1** | **ZNF550** |
| **HOXC12** | **NR2F1** | **SAP18** | **FOXJ2** | **ZNF342** |
| **HOXC13** | **NR2F2** | **SNAPC5** | **HDAC8** | **ZNF564** |
| **HOXD1** | **TFDP1** | **ZNF267** | **ASH1L** | **ZNF709** |
| **HOXD4** | **TFDP2** | **ZNFN1A1** | **LMO3** | **ZNF383** |
| **HOXD8** | **TGFB1I1** | **TRIM22** | **ZNF167** | **ZNF100** |
| **HOXD11** | **TGIF** | **NPM2** | **MYNN** | **ZNF540** |
| **HOXD12** | **THRB** | **HMG20B** | **ZNF302** | **CITED4** |
| **HOXD13** | **KLF10** | **HMG20A** | **MLL5** | **ZNF366** |
| **HES1** | **TIAL1** | **KLF2** | **BARX1** | **ZNF679** |
| **HSBP1** | **TITF1** | **CITED2** | **NDNL2** | **BHLHB8** |
| **HSF1** | **TLE1** | **ISGF3G** | **ZNF253** | **ZNF596** |
| **HSF2** | **NR2E1** | **SCML2** | **CTNNBL1** | **GLIS3** |
| **HTLF** | **TMF1** | **PIAS3** | **ANKRD7** | **MGC17403** |
| **IRF8** | **TP53** | **C1D** | **PPAN** | **GSH2** |
| **ID2** | **NR2C1** | **ZNF238** | **ASCL3** | **ZNF431** |
| **ID3** | **NR2C2** | **HMGN4** | **SLC2A4RG** | **ZNF384** |
| **ID4** | **TRIP6** | **TADA3L** | **BARHL1** | **POLR3H** |
| **SP110** | **TWIST1** | **HOXB13** | **PAPOLB** | **FLJ31875** |
| **IGHMBP2** | **UBTF** | **CREB3** | **C21orf7** | **ZNF553** |
| **RBPSUH** | **NR1H2** | **MYBBP1A** | **MEIS3** | **ZNF627** |
| **IKBKB** | **USF1** | **ZNF211** | **LHX9** | **FOXD4L1** |
| **ILF2** | **USF2** | **HTATIP** | **PRDM9** | **ZSCAN4** |
| **ILF3** | **VDR** | **BATF** | **PRDM10** | **TIGD4** |
| **INSM1** | **VHL** | **HTATIP2** | **CTNNBIP1** | **KHDRBS2** |
| **IPF1** | **WHSC2** | **DRAP1** | **TBX20** | **ZNF449** |
| **IRF1** | **WT1** | **MXD4** | **REXO4** | **LASS3** |
| **IRF2** | **XBP1** | **HEXIM1** | **INTS12** | **GSH1** |
| **IRF4** | **XRCC5** | **C20orf18** | **ZNFX1** | **ZNF25** |
| **IRF5** | **YY1** | **POLR3F** | **ZNF286** | **ZBTB9** |
| **ISL1** | **ZFP37** | **POLR3C** | **ZNF287** | **ZBTB12** |
| **JUN** | **ZFP161** | **IVNS1ABP** | **ZNF304** | **tcag7.981** |
| **JUND** | **ZFX** | **TRIM16** | **GATAD2B** | **RFXDC1** |
| **LMO1** | **ZFY** | **TAF6L** | **CNOT6** | **ZNF390** |
| **LMO2** | **ZIC1** | **RGS14** | **GM632** | **VGLL2** |
| **LMO6** | **ZNF2** | **KHDRBS1** | **ZNF490** | **CNOT6L** |
| **MXD1** | **ZNF3** | **LBX1** | **ARID1B** | **POLR2J2** |
| **SMAD1** | **ZNF6** | **KLF1** | **MKL2** | **ZNF396** |
| **SMAD2** | **ZNF9** | **CTCF** | **MTA3** | **NEIL2** |
| **SMAD3** | **ZNF10** | **GMEB1** | **ZNF398** | **ZBTB38** |
| **SMAD4** | **ZNF11B** | **TBR1** | **ZNF624** | **EBF3** |
| **SMAD5** | **ZNF12** | **TCFL5** | **ZNF471** | **MLR1** |
| **SMAD6** | **ZNF16** | **RAI1** | **RP5-860F19.3** | **MCMDC1** |
| **SMAD7** | **ZNF17** | **JARID1B** | **KIAA1443** | **BCL6B** |
| **SMAD9** | **ZNF20** | **ZMYND11** | **ZBTB2** | **PTF1A** |
| **MAF** | **ZNF21** | **FUSIP1** | **POGK** | **ZNF311** |
| **MAFG** | **ZNF22** | **ZBTB6** | **PHF12** | **MKX** |
| **MAX** | **ZNF23** | **ZNF271** | **KIAA1559** | **PTRF** |
| **MBD1** | **ZNF26** | **ZNF234** | **ZBTB26** | **ZNF547** |
| **MCM2** | **ZNF27** | **ZNF266** | **NCOA5** | **ZSCAN1** |
| **MCM5** | **ZNF29** | **ZNF273** | **RBAK** | **ZNF283** |
| **MDS1** | **ZNF32** | **ZNF272** | **SF4** | **ZNF615** |
| **MECP2** | **ZNF33A** | **ZNF275** | **GATAD1** | **ZNF545** |
| **MEF2C** | **ZKSCAN1** | **RUVBL2** | **ZNF410** | **LOC115648** |
| **MEIS2** | **ZNF37A** | **PPARGC1A** | **ZF** | **ZNF493** |
| **MEIS3P1** | **ZNF38** | **SUB1** | **ZNF77** | **HKR1** |
| **MEN1** | **ZNF41** | **SFRS2B** | **ZNF250** | **ZNF454** |
| **MEOX2** | **ZNF42** | **MORF4** | **SR-A1** | **ZFP41** |
| **CIITA** | **ZNF43** | **MSL3L1** | **MLL3** | **RP11-561O23.3** |
| **MITF** | **ZNF45** | **COPS5** | **PRDM12** | **SALF** |
| **MLL** | **ZBTB25** | **ATF7** | **PRDM13** | **SNAI3** |
| **MLLT1** | **ZNF70** | **RY1** | **TGIF2** | **CHX10** |
| **AFF1** | **ZNF74** | **ALF** | **ALX4** | **ZNF546** |
| **MLLT7** | **ZNF75** | **WWP1** | **ANKRD5** | **ANKRD45** |
| **NR3C2** | **ZNF75A** | **CEP110** | **DMRTC2** | **ZDHHC21** |
| **MNDA** | **ZNF79** | **TRIM31** | **DMRTB1** | **ANKRD33** |
| **MNT** | **ZNF80** | **HSF2BP** | **NEUROG2** | **ZNF677** |
| **CITED1** | **ZNF84** | **HNRPUL1** | **NEUROD6** | **HKR2** |
| **MYB** | **ZNF85** | **PRDM4** | **FKSG14** | **BARHL2** |
| **MYBL1** | **ZNF90** | **SFRS16** | **MMS19L** | **FIGLA** |
| **MYCL1** | **ZNF91** | **MYST2** | **ZNF323** | **ZFP57** |
| **MYCN** | **ZNF222** | **SOX21** | **HIF3A** | **ZNF81** |
| **MYF5** | **ZNF121** | **WDHD1** | **POLR1E** | **TBX10** |
| **MYF6** | **ZNF124** | **BAZ1A** | **ZDHHC6** | **ZNF713** |
| **MYT1** | **ZNF131** | **LZTS1** | **EBF2** | **FOXD4L4** |
| **NAB1** | **ZNF132** | **ZNF277** | **SMURF2** | **ZNF445** |
| **NAB2** | **ZNF133** | **TNRC4** | **ISL2** | **LOC360030** |
| **NAP1L3** | **ZNF134** | **SUPT16H** | **RFXDC2** | **ZNF307** |
| **NAP1L4** | **ZNF136** | **DDX20** | **PAPOLG** | **MAFA** |
| **NEUROD2** | **ZNF137** | **POU6F2** | **C2orf26** | **ZBTB34** |
| **NEUROG1** | **ZNF138** | **RBPSUHL** | **ZNF447** | **BOLA2** |
| **NFATC3** | **ZNF140** | **PHB2** | **ZBTB10** | **LOC643641** |
| **NFATC4** | **ZNF141** |  |  |  |
